# Supplementary material for: Patterns of Intron Gain and Loss in Fungi
Source: PLoS Biol. 2004 Nov 30;2(12):e422. doi: 10.1371/journal.pbio.0020422 (PMC532390; doi:10.1371/journal.pbio.0020422)
Supplement: Table S1 — Also available at http://genes.mit.edu/NielsenEtAl/. (4.3 MB ZIP). [file pbio.0020422.st001.zip › NielsenEtAl/html/378.html]

AN3895.1.NCU00052.1.MG03774.1.FG03666.1


```
 CLUSTAL W (1.82) Multiple Sequence Alignments - Introns Inserted


Sequence 1: MG03774.1	552 aa
Sequence 2: FG03666.1	565 aa
Sequence 3: AN3895.1	570 aa
Sequence 4: NCU00052.1	640 aa
Alignment Length: 658 aa
Number Identitical Residues: 203 aa
Alignment Score (without introns) 10994


MG03774.1 	--MVLHTRYSATAESWRLLDLVLDSFASDQLSLPTAARLRAKSNVGFCAS--RDFPYFPI
NCU00052.1	MASSATRPYSVAAGASEAFGELLKQC---KGQVPKEFGSYSGN-VTFVPSNHGDQVYFPS
FG03666.1 	MGSISHSGYSSVEESKRIFNYLCDQF--DETALPAGVKELRDS-IEFSSS--RDAPYFPI
AN3895.1  	-----MASYSPVKEAMRIFSFLCDQA--ERLNLPPEVVAGQDA-VSFNTT--HDKIYFPI
          	        ** .  : . :. : ..   ..  :*          : * .:   *  *** 

MG03774.1 	PFKETETTAVLKAVEAAVAAAILDARNG-VDVERRITIDLERTTAFLFQAYLATVGGLGK
NCU00052.1	PCREQDAIAAIKALEACAAAAIGDLRHG--KNSRSIKVDLDKAACFLMSAYLATIDGKRK
FG03666.1 	PFKETETTAALKAVEGAVACLLAGSAAG-ESQTRKMNINLEKTTAFLCQAYMAKVGGLGK
AN3895.1  	PFKETETLAALKGVEGSVAAAIADLRYGPAAQKRNIKINLERATAFGCQAYMAKVDGMSK
          	* :* :: *.:*.:*...*. : .   *.    * :.::*::::.*  .**:*.:.*  *

MG03774.1 	LDP--------~-~------E0SDPYRRMSANLYETKRPGEYYHIHGSLEASTTLGMIGL
NCU00052.1	GQTPKGLIP--~D1TDFNQAQ~SIQYRRLSANLYETKEDGRYYHIHGSLDATTQLRMIGL
FG03666.1 	LDPGVKAML-K~D1TDLLQAQ~SNQYRRMSANLYETKNPGEYYHIHGSLEASTTLKMIGL
AN3895.1  	LDPEVKKKLKV1D~TDLLAAQ~SNGYRRMSANLYRTKNEGEFFHIHGSLEATTTLNMIGL
          	 :.      .  . :.   :: *  ***:*****.**. *.::******:*:* * ****

MG03774.1 	EPFRPDLHTHEEISSTIEPAVKQFTIEELEQMNAKNRQAGVPVLKHEDFLKTPH0GQVMS
NCU00052.1	LPFNPRLTDYQKCIDTIEAAVKRFTVAELEDMNSKHGQAGVEALKWENFLETTH~GKVIS
FG03666.1 	EPYRPDLQTHESIVDTIEPAVRKFSIEELETMNAANRQAGVPALKHEDFVKTEH0GKTNM
AN3895.1  	EGHRPDLTDYEEIIKVIESHVQKYSAAELEEMNNERRQAGVTAFKYEDFIKTPH0GKLNV
          	  ..* *  ::.  ..**. *::::  *** **  . **** .:* *:*::* * *:   

MG03774.1 	KSAPWSVIPLEESTPPCPLP-----------------AAKD--NRPLSGIKVLEMCRIIA
NCU00052.1	ALPPFTVEAIKVQQTTVPETPPHTPTETKFQSHLWPKVPANVSQYALRGIKVIEMCRVIA
FG03666.1 	ALPPWSVENLESETPKCPLN-----------------QTSEQPKRILSGIKVLELCRIIA
AN3895.1  	QQPPWKVTKLPGNLPPTPFP-----------------ADGGN-KKILKGIKVLELCRIIA
          	  .*:.*  :  . .  *                         :  * ****:*:**:**

MG03774.1 	GPTIARILAEYGATVLKITSPNLSDVPFFQVDGNMGKHAADLDLKTPAGRTEFEKLVADV
NCU00052.1	GPTIGRSLAAHGAQVLKVTGPKLPDVPFFQVDVNTGKHTSHLDLRNQSDLKTFQNLLAEA
FG03666.1 	GPIITRILGEYGADVLKITSPNLSDVPFFQVDGNMGKHAAELDLKSAEGRAKFEVLLAEA
AN3895.1  	GPTVARILTEYGADVLKITSPHLSDVPFFQVDGNMGKHAADLDLKTPEGREVFEALLADA
          	** : * *  :** ***:*.*:*.******** * ***::.***:.  .   *: *:*:.

MG03774.1 	DIVVDGYRPGALEKLGYGPEALSRLAKERG-KGVVYVNENCFG----YEGEWAHRPGWQQ
NCU00052.1	DVLIDGYRPGALAKFGCDPQSLAKLAQQRGNHGFVYVAEDCFGGTGVPGAEWAGRRGWQQ
FG03666.1 	DVVVDGYRPGALEKLGYGATALTKLAKERG-KGFVYVNENCFG----YQGEWAGRPGWQQ
AN3895.1  	DVLVDGYRPGALDRLGYGASALAALAEKRG-KGIVYVNENCFG----YEGEWAGRPGWQQ
          	*:::******** ::* .. :*: **::** :*.*** *:***      .*** * ****

MG03774.1 	IADC0VTGIAWEQGRFMGLS-EPVVPPFPISDYGTGCMGAIAALAGLLNRTSRGGSWHGR
NCU00052.1	IADC~VTGVAWAQGQFMGLENEPSVPPMPMSDYGTGALGCVAAMAGLYQRETKGGSWMCR
FG03666.1 	IADC0VTGIAWEQGRFMGMS-EPMVPPFPISDYGTGCMGAITALIGLYHRATKGGSWHGK
AN3895.1  	IADC0VTGVAWAQGKFMNLP-TPVVPPFPISDYGTGCMGAIAALTGLYNRAKFGGSYHGK
          	**** ***:** **:**.:   * ***:*:******.:*.::*: ** :* . ***:  :

MG03774.1 	ASLVQYDMLLFRAGAYPDDVVRKLREESGRWAGFDALRHDNSVDQISGTALRGMRERFPE
NCU00052.1	TSLCQYDIFLLKLGLLPLQEQERLKMVFAG--PFFKLRHYDSVDKVSGEALKAMQRAYPH
FG03666.1 	ASLLHYDLLLFKVGQYPEEIKERLRKEIG--PDFLALRHAHSVDQISGTALLKMRDTYPE
AN3895.1  	ASLMHYDLLLFAVGQYDQDVQESLRASQP--EEFFSLRHCDSVDRISSTVLKGMKERFPH
          	:** :**::*:  *    :  . *:        *  *** .***::*. .*  *:  :*.

MG03774.1 	LFA---RRD-----LTDCWFSARYGAEVRAVAPVVQIEGAEVSFSRASRPNGSDPA---K
NCU00052.1	LFG---PDL-----MLKAKSKGFNGKDIRWPKEAIEVEGLLIQHVRASRPNGFDRPGWEG
FG03666.1 	LFS---SDK-----YVEKWYSNAYKAEVEAVPPVVEIEGIDVGFRRASRPNGADEP---T
AN3895.1  	LYLPPGSDKEHEVILTEKWYSTAYGAEIEVVKPIAEIDGVDNSFERASRPNGVDRP---S
          	*: ...   . .    .   .     ::.      :::*    . ******* * .    

MG03774.1 	WEFG--DHGDYRKEA---------------------------------------------
NCU00052.1	WETDLIMDGEVNLAEDDAVFTNTSVTPAPREEQHQQQAGSGSMNSVPQRRNRIATMNTVL
FG03666.1 	WDFG--HEPDHKLSK---------------------------------------------
AN3895.1  	WEDFRVEEGDKRLQ----------------------------------------------
          	*:     . : .                                                

MG03774.1 	---
NCU00052.1	ATA
FG03666.1 	---
AN3895.1  	---
          	
```
